# Insecticidal toxicity of essential oil of Nepalese *Acorus calamus* (Acorales:Acoraceae) against *Sitophilus zeamais* (Coleoptera:Curculionidae)

**DOI:** 10.1016/j.heliyon.2023.e22130

**Published:** 2023-11-10

**Authors:** Sunil Aryal, Ashmita Poudel, Kapil Kafle, Lok Nath Aryal

**Affiliations:** a Horticulture Research Station, Pokhara, Kaski, Nepal Agricultural Research Council, Nepal; b Tribhuvan University, Institute of Agriculture and Animal Sciences, Kirtipur, Kathmandu, Nepal

**Keywords:** Sweet flag, Maize weevil, Oil extraction, Concentrations, Management

## Abstract

Maize weevil (*Sitophilus zeamais)* (Coleoptera:Curculionidae) is an economic stored grain pest that causes significant damage to various stored products, including maize (*Zea mays*). In this study, we extracted essential oil from the rhizome of sweet flag (*Acorus calamus)* (Acorales:Acoraceae) by hydro-distillation and tested insecticidal property of the oil at 7 concentrations (10, 5, 2.5, 1.25, 0.625, 0.3125, 0.15625 and control) against maize weevil (*Sitophilus zeamais*) at the National Entomology Research Center, Nepal Agricultural Research Council in the year 2020/2021. Three different experiments were conducted: scintillating vial bioassay, repellency test, and exposing weevils to oil treated maize grains. Scintillating vial bioassay showed that higher the concentration of essential oil, lower the time required to cause 50 % maize weevil mortality. Median lethal concentration (LC_50_) at 3 and 24 h was calculated as 2.29 and 0.16 % of oil concentration in scintillating vial bioassay. When oil is treated to maize grain, LC_50_ for 10 and 16 days was calculated as 2.77 and 0.23 % of oil concentrations. In the same way, at 10 % concentration maize weevil showed highest repellent activity (98.75 %) as compared to 5, 2.5 and 1.25 % concentrations after 24 h of treatment. Weight loss and grain damage were significantly less in the oil treatments than the control. However, from the perspective of health benefits, *Acorus calamus* treated maize is still questionable for feed and food purpose. As β asarone has carcinogenic effects at certain level, it needs further residue tests of treated maize to know allowable maximum residue limit (MRL) before consumption as food or feed.

## 1 Introduction

Storage pests have economic importance as they inflict considerable losses to grain which is acquired with lots of efforts in the field, if grains are not properly handled and stored. The maize weevil (*S*. *zeamais*) is one of the most common storage pest of maize in Nepal. One study revealed about 10–20 % of the grain losses in storages of Nepal is due to insect pests [1]. This coleopteron insect feeds inside the kernels causing quality and weight loss [2], which hinder proper germination losing its seed value [3]. This weevil causes the loss of up to 80 % in tropical countries [4,5], whereas in Nepal losses due to this insect ranges from 18 to 40 % [6]. One of the primary method to control insect pests of grains during storage is chemical pesticides [7]. Though pesticides treatment to grains seems effective and easy to use, it may cause toxic effects for humans, the environment and not-target organisms. Pesticide contaminated grains with high residues will have significant impact on human and animal health if consumed without maintaining proper waiting periods.

Plant products are used to extend the life of stored products since ancient time [8]. Sweet flag (*A*. *calamus*) of the genus *Acorus* belongs to the family Acoraceae and is extensively studied due to its medicinal and pharmacological properties [9,10]. This plant species grows near swamps, rivers, lakes and marshy places up to 2000 m altitude in different parts of Himalayan and sub-Himalayan regions of Nepal and India [[11], [12], [13], [14]]. This plant has also importance in food, cosmetic and perfume industries [15]. Furthermore, rhizomes of *A*. *calamus* possess antibacterial and antifungal properties [16,17]. This plant contains bioactive chemical constituents like alkaloids, steroids, phenols, coumarins, flavonoids etc. which are valuable for controlling insect pests [[17], [18], [19], [20]]. Among the several phytochemical constituents, the major acting against insect pests are β-asarone and shybunone, which are present in various concentrations [[21], [22], [23], [24]]. The major constituents of essential oil of diploid North American *A. calamus* is shyobunones (13–45 %) while tetra or hexapliody East Asian *calamus* contains β-Asarone (42.5–78 %) [[25], [26]]. The major components of the essential oil used in these studies were β asorone, Dehydroxy-isocalmendiol, Shyobunone, Cuparene, Epishyobunone, Isoshyobunone and δ-Cadinene which constitute about 79.7, 4.03, 3.27, 0.93, 0.68, 0.47 % respectively [27]. There were other compounds detected in minor quantities. Another Nepalese *A. calamus* consists of β-methyl isoeugenol, Caryophylene oxice, 6-Epishyobunone, β-Isoelemicin, β-asarone, alpho-asarone etc where β-asarone constitute about 84.87 % [28]. As β-asarone and shyobunone are the major compounds which is effective against pests, the essential oil used in this study could be potentially effective as it was evident by other studies too.

When tested in rats, *A. calamus* acted as sedative, CNS depressant, behavior modifier, anticonvulsant, acetcholinesterase inhibitory, genotoxic and mutagenic [29]. Essential oil actions which were mostly discussed are the inhibition of acetylcholinesterage and it also interfere with octopanime binding with its receptor [30]. Essential oil further has effects on plasmocytes and granular hemocytes of insects. The cause of death of the insects from *A. calamus* essential oil may be due to the insect's inability to detoxify β-asarone which inhibit P450 genes [31]. Similarly, genetic toxicity has been reported by Kumar et al. [32] where damage of gene is the cause of mortality in *Dorsophila melanogaster*. Moreover, *A. calamus* oil caused agglutination and immobility of sperm which ultimately make male sterility and morphologically deformed female tracts [33].

*A. calamus* rhizome powder has a good grain protectant against *S*. *oryzae, S. granaries* and *S*. *zeamais* [25]. If admixed with grains, it can be effective for five [34] to seven months with negligible damage to the grains. Use of powder of *A. calamus* of Nepalese origin has been studied extensively but the study on oil of same against insect pests is very limited. Therefore, this study aims to establish median lethal concentration of essential oil from *Acorus calamus* rhizome, and its repellent effect to control weevil's damage.

## 2 Materials and methods

### 2.1 Insect rearing

The maize variety Manakamana- 4 was used for the insect rearing. Grain moisture was adjusted to 14 % to make favorable condition for insect growth and development. Amount of water to be added to adjust moisture to desired level is given by Adams and Schulten [35].

$$\mathrm{Weight}\;\mathrm{of}\;\mathrm{water}\;\mathrm{to}\;\mathrm{be}\;\mathrm{added (g)}=weight\;of\;grain\times \frac{Desired\;moisture\;\%-Present\;moisture\;\%}{100-\text{Desired}\;\text{moisture \%}}$$

Sterilized maize (500 g) for 30 min was kept in 1 L capacity glass jar. Weevils was obtained from National Entomology Research Center (NERC), Nepal Agricultural Research Council (NARC). One hundred weevils were added in a clean and sterilized 1 L jar. The open part of jar was covered with black colored muslin cloth and made tight using rubber band. Eight glass jars of similar shape and size were used on weekly basis to regularly supply weevils for the experiment. Glass jar ready for rearing was kept in a laboratory conditions. Daily temperature and humidity of laboratory were recorded hourly using data logger. After 7 days the adult weevil were removed from the glass jar with feather touch forceps. Average temperature and relative humidity at rearing room during the study period of six months was 23.7 ± 0.1 °C and 68.7 ± 0.3 %, respectively.

### 2.2 Oil extraction

Rhizomes of *A. calamus* were collected digging from cultivated field of NERC, NARC, Khumaltar. It was cleaned using tap water, chopped into pieces, and then left for drying in room under shaded condition for about 15 days. The shade dried rhizomes of *A. calamus* were powdered. A hundred grams of the powdered sample was hydro-distilled for 4 h at 60 °C using Clevenger apparatus [36,37]. Glass beads (7–8) were added in the flask to prevent the bumping of the liquids. Extracted essential oil was dried using anhydrous sodium sulphate and kept in refrigerated condition at 4 °C in amber color vials. Amount of oil obtained from 100 g of powder after distillation was 1.67 g.

### 2.3 Toxicity evaluation

#### 2.3.1 Scintillating vial bioassay

Seven different concentrations were prepared from the extracted oil of the *Acorus calamus* with methanol starting from 10 % as a stock solution. Further dilution was made with dilution factor of 2 which comprises of further 6 solutions of 5, 2.5, 1.25, 0.625, 0.3125 and 0.15625 %. The inner surface of the vial and lid was coated with 300 μL of each of the 7 concentrations by rolling to make sure that the entire surface inside the vial and lid was contaminated. Four controls were also maintained coated only with methanol. Vials were kept for drying at room condition until the solvent used was evaporated. After drying, 15 adult maize weevils were placed inside the vials. Four vials for each concentration were maintained. No food was provided. The first two observations were made on 3 and 12 h of treatment. The rest of the observations were taken at 24 h’ interval until all the weevil died in treated vial, which was 144 h of the weevils inoculation. Weevils were prodded with camel hair brush and if no movement was observed, they were regarded as dead. To confirm death, weevils were often observed under stereomicroscope (SZ61, Olympus). Average temperature and relative humidity at experiment room during the study period was 25 °C ± 1 °C and 65 ± 5 % respectively. The seven concentrations and one control (methanol only), four replications were designated to completely randomized design (CRD) in laboratory.

#### 2.3.2 Treated maize bioassay

Fifty grams of sterilized maize at 100 °C for 30 min was kept in a 100ml jar and moisture was adjusted to 14 % as described in insect rearing section. The maize variety used in the experiment was Manakamana-4. Seven dilutions were made from 10 % of stock solution as in case of scintillating vial experiment. A Half ml of each concentration was added in each jar. The control was made only by adding methanol. The lid of jar was closed and shaken for uniform coating of the oils to the grains. Twenty unsexed adults of 7–14 days old weevils were released at the center of plastic jar. Then the jar was covered with perforated lid tightly and kept at laboratory condition. Mortality of weevil were assessed every day (24 h interval) up to 10 days and at 12, 16 and 24 days of weevil inoculations. The number of seeds damaged, seed weight loss and progeny emergence were also recorded after 60 days of weevils’ inoculation. Each concentration was replicated four times.

#### 2.3.3 Seed damage and weight loss

Sixty days after the introduction of adult weevils, the number and weight of damaged and undamaged seeds were recorded. Damages were assessed by visual observation. If required, hand lenses were used. Seeds were considered damaged even small exit hole was made in grain by weevil. Seed weight loss was calculated by using the count-and-weight method.

#### 2.3.4 Progeny development

The number of F1 progeny weevils emerging was recorded up to sixty days by which all the progeny had emerged. Emerged adults were removed from the jar on assessment day.

#### 2.3.5 Repellency effect

Repellency assays of essential oil were carried out according to the experimental method described by Kafle and Shih [38] at 25 °C ± 1 °C and 65 ± 5 % RH. Petri dishes (Ǿ 8 cm)were washed with detergent water and rinsed with distilled water and dried in oven. Whatman filter papers (Ǿ 8 cm) were fold and marked in half and placed properly in petri dishes. Required concentration of test solutions were prepared by extracted oil with methanol which comprised of solutions of concentration 10, 5, 2.5, and 1.25 %. Solution of each concentration (0.5 ml) was applied to half a filter paper disc uniformly with micropipette. The other half of the filter paper was treated with methanol only as a control. Twenty unsexed adults of 7–14 days old weevils were released at the center of the filter paper. The insect present at treated and untreated area of filter paper were counted after 24 h. Each concentration and control was repeated four times.

#### 2.3.6 Germination test

The effect of treatments on seed germination and viability was examined after 60 days of grain storage period. Seed germination was tested using 10 randomly picked seeds from undamaged grains after separation of damaged and undamaged grains in each jar according to the methods described by Haines [39]. Small tea glass sized plastic jars (250 ml) were taken for the experiment. Small holes were made to prevent water logging. Plastic jars were placed at outdoor condition. Soil mixed with sand was filled in the plastic jar. The 10 grain sub-samples were germinated in plastic jar in a Randomized Complete Block Design with four replicates. The number of germinated seedlings from each Petri dish was counted and recorded after 7 days. Viability was computed as the percentage of germinated seeds divided by the total number of seeds. Average maximum temperature, Minimum temperature, relative humidity, and hourly average solar radiation during the experiment period was 25.44 °C (±0.24), 8.8 °C (±0.39), 54.5 % (±0.40) and 163.20 W/m^2^ (±17.4) respectively.

### 2.4 Statistical analyses

The data from all the experiments were recorded and managed in a MS- excel sheet. The SPSS (16th version) was used for data analysis [40]. The concentrations of each essential oil were transformed using log transformation at the base of 10. Data were subjected to probit analysis [41]. A chi-square test was used to test for heterogeneity within the data. If heterogeneity was significant (p < 0.05) a heterogeneity factor was included to calculate variances and confidence limits. Probit regression analysis was done to calculate LC_25_, LC_50_ and LC_90_ of essential oil of Sweet Flag to maize weevil at 6, 10 and 16 days in scintillating vial bioassay. Analysis of variance (ANONVA) was performed to see the repellency effect of *A. calamus* essential oil. Mean damage, weight loss, progeny number, and germination percentage were analyzed with ANOVA and means were compared with Tukey's test at 0.05 %.

## 3 Results

### 3.1 Scintillating vial bioassay

**Fig. 1:**
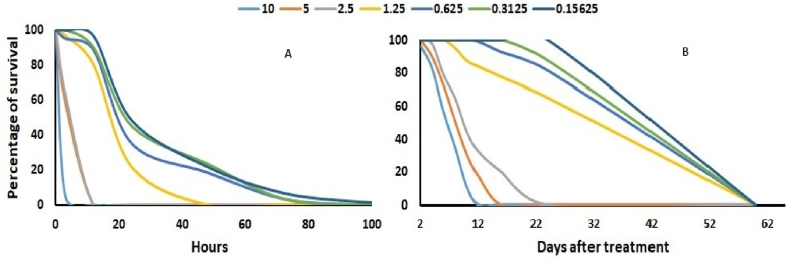
Survival of *Sitophilus zeamais* to different concentration of essential oil in (A) Scintillating vial bioassay and (B) Treated maize bioassay.

At 3 h, probit regression estimated 2.92 % concentration of essential oil of sweet flag is required for the 50 % mortality of weevil population, likewise to kill 90 % population in 3 h, concentration of essential oil required was 8.75 % (y = 2.684x-1.247). Whereas at 24 h, the concentration required to kill 50 % decreased up to 0.16 % oil and only 1.151 % concentration was enough to cause 90 % mortality (y = 1.51x+1.19) (Table 1). As the concentration of oil decreases, time required to cause mortality increases (Fig. 1A) where only 0.54 h required for 10 % oil where as it was 27.67 h when concentration used was reduced to 0.15 % (Table 2).

**Table 1:** LC_50_ and LC_90_ (%) of the essential oil extract of sweet flag in scintillating vial bioassay for mortality of *Sitophilus zeamais* at 3 and 24 h.

Hrs	LC_50_ (CL_95_ %)	LC_90_ (CL_95_ %)	*χ*^2^	slope (±SE)	intercept (±SE)	P
3	2.92 (2.26–2.94)	8.75 (5.95–7.19)	66.12	2.68 (±0.25)	−1.24 (±0.13)	<0.001
24	0.16 (0.11–0.21)	1.15 (0.85–1.73)	37.64	1.51 (±0.17)	1.19 (±0.10)	<0.001

n = 60 in each concentration, LC = Median lethal concentration, CL=Confidence intervals.

**Table 2:** LT_50_ of the essential oil extract of Sweet flag in scintillating vial bioassay tested for mortality of *Sitophilus zeamais* with different seven concentrations.

Concentration (%)	LT_50_ (CL_95_ %) (Hours)	*χ*^2^	Slope (±SE)	Intercept (±SE)	p
10	0.54 (0.01–1.26)	59.08	2.15 (±0.55)	0.58 (±0.31)	<0.001
5.0	3.33 (2.77–4.10)	186.14	4.70 (±0.45)	−2.46 (±0.24)	<0.001
2.50	3.55 (3.28–3.87)	43.02	4.69 (±0.39)	−2.58 (±0.21)	<0.001
1.25	15.66 (10.54–21.27)	845.51	3.97 (±0.16)	−4.74 (±0.21)	<0.001
0.63	20.06 (16.27–24.04)	265.93	2.98 (±0.09)	−3.86 (±0.14)	<0.001
0.31	25.04 (22.68–27.43)	94.61	3.77 (±0.13)	−5.28 (±0.19)	<0.001
0.16	27.67 (22.34–33.18)	407.53	3.93 (±0.13)	−5.67 (±0.21)	<0.001

n = 60 in each concentration, LC = Median lethal concentration, CL=Confidence intervals.

### 3.2 Treated maize bioassay

After 6 days, 0.25, 2.89 and 211.63 % concentration of essential oil was required to kill 25, 50 and 90 % of weevil, respectively (y = 0.68x-3.17). Likewise, 0.07, 0.26 and 2.77 % concentration was required to kill 25, 50 and 90 % of population at 10 days, respectively (y = 1.24x+0.73). After 16 days, 0.04, 0.07 and 0.23 % concentration of oil was enough to kill 25, 50 and 90 % of weevil population (y = 2.54x+2.92) (Table 3).

**Table 3:** LC_25_, LC_50_ and LC_90_ (%) of the essential oil of sweet flag treated maize bioassay tested for mortality of *Sitophilus zeamais* at 6, 10, 16 days.

Days	LC_50_	LC_25_	LC_90_	*χ*^2^	Slope (±SE)	Intercept	P
6	2.89 (2.42–3.52)	0.25 (0.23–0.30)	211.63 (120.8–429.06)	26.01	0.68 (±0.42)	−3.17 (±0.25)	<0.001
10	0.26 (0.21–0.3)	0.07 (0.05–0.09)	2.77 (2.26–3.53)	46.72	1.24 (±0.05)	0.73 (±0.30)	<0.001
16	0.07 (0.05–0.08)	0.04 (0.02–0.05)	0.23 (0.2–0.25)	16.04	2.54 (±0.27)	2.92 (±0.19)	<0.001

n = 80 in each concentration, LC = Median lethal concentration, CL=Confidence intervals.

Median lethal time for 50 % mortality of *S*. *zeamais* with sweet flag essential oil 10 % concentration is 3.8 days (y = 3.38x-1.96), whereas lethal time for 50 % mortality at 5 % concentration is 5.25 days (y = 4.03x-2.9). Likewise, at 2.5 % concentration, 5.87 days was required for the 50 % mortality of population (y = 4.53x-3.48). Further, 6.21 (y = 4.41x-3.5), 7.8 (y = 4.33x-3.86), 9.91 (y = 4.31x- 4.15), 10.03 (y = 4.58x-4.59) days are required to kill 50 % of the weevil population at 1.25, 0.625, 0.31 and 0.156 % concentration of essential oil, respectively (Table 4, Fig. 1B).

**Table 4:** LT_50_ of the essential oil extract of Sweet Flag (*Acorus calamus*) in treated maize bioassay tested for mortality of *Sitophilus zeamais* with different seven concentrations.

Concentration	LT_50_ (days)	*χ*^2^	Slope (±SE)	Intercept (±SE)	P
10	3.809 (3.52–4.09)	233.47	3.38 (±0.86)	−1.96 ± 0.64	<0.001
5	5.25 (4.87–5.64)	374.18	4.03 (±0.10)	−2.9 (±0.83)	<0.001
2.5	5.87 (5.62–6.11)	152.58	4.53 ± (0.11)	−3.48 (±0.98)	<0.001
1.25	6.21 (5.98–6.44)	118.18	4.41 (±0.12)	−3.50 (±0.09)	<0.001
0.63	7.8 (7.5–9.09)	114.06	4.33 (±0.11)	−3.86 (±0.10)	<0.001
0.31	9.19 (8.85–9.56)	120.63	4.31 (±0.11)	−4.15 (±0.11)	<0.001
0.16	10.03 (9.72–10.36)	83.16	4.58 (±0.13)	−4.59 (±0.12)	<0.001

n = 80 in each concentration, LT = Median lethal time, CL=Confidence intervals.

### 3.3 Repellent effect

After 24 h of treatment, significantly more weevils were present on the untreated half of the petridishes whereas sweet flag oil-treated half of the petri dishes at all the concentration have less weevil present. At the treated half (F_3,16_ = 15, p < 0.001) and untreated half (F_3,16_ = 18.02, p < 0.001) of the Petri dishes there is significant difference on the presence of adult weevil among concentrations (Table 5).

**Table 5:** Repellency of sweet flag oil representing the percentage of adults of *Sitophilus oryzae* adults present on treated and untreated are after 24 h.

	Adult *S. oryzae* weevil percentage	
Concentration %	Treated area	Untreated area
10	1.25 (±1.08)^c^	98.75 (±1.08)^a^
5	6.25 (±2.07)^bc^	93.75a (±2.10)^a^
2.5	16.25 (±2.07)^ab^	82.5b (±1.25)^b^
1.25	23.75 (±3.25)^a^	76.25 (±3.25)^b^
F value	15.0	18.02
P	<0.001	<0.001
Df	3,16	3,16

### 3.4 Progeny emergence

Progeny development was significantly different among treatments (F_7, 32=_314.22, P= <0.001). No progeny emergence was observed in all treated maize. Live progeny (no. 72) was observed only in untreated maize. (Table 6).

**Table 6:** Mean damage, weight loss, progeny number, and germination percentage on maize treated with *A. calamus* essential oil.

Concentration	Wt. loss (g)	Damage %	Progeny (number)	Germination %
10	0.715^a^	0.56 (±0.01)^a^	0^a^	95 (±2.9)^a^
5	1.17 (±0.13)^a^	0.56 (±0)^a^	0^a^	87.5 (±2.50)^ab^
2.5	1.62 (±0.09)^ab^	1.27 ((±0.27)^a^	0^a^	85 (±2.89)^abc^
1.25	2.38 (±0.06)^bc^	2.44 (±0.38)^a^	0^a^	77.5 (±2.5)^bcd^
0.63	3.2 (±0.24)^c^	2.75 (±0.37)^a^	0^a^	72.5 (±2.5)^cde^
0.31	5.2 (±0.14)^d^	3.88 (±0.18)^a^	0^a^	65 (±2.89)^de^
0.16	6.42 (±0.06)^e^	14.71 (±0.67)^b^	0^a^	60 (±0)^e^
Control	24.78 (±0.58)^f^	59.38 (±2.09)^c^	72 ± 2.44^b^	30 (±4.08)^f^
df	7, 32	7, 32	7, 32	7, 32
F value	11.20	627.15	314.22	55.31
P value	<0.001	<0.001	<0.001	<0.001

### 3.5 Damage and weight loss percentage

Weight loss in grain treated with 10 % and 5 % concentration of essential oil of *A. calamus* was significantly low compared to other concentrations which was similar with grain treated with 2.5 % concentration (F_7,32_ = 11.20, p < 0.001) (Table 6). Higher weight loss (24.78 %) was observed in control treatment. Lower weight loss (0.71 %) was observed in treatment with 10 % concentration compared to the control (24.7 %).

Grain treated with 10 % concentration of essential oil has lowest damage, which was statistically similar with 5, 2.5 %, 1.25 %, and 0.31 % concentration (F_7, 32_ = 627.15, p < 0.001). Damage percentage also differed among the oil concentrations. Maximum damage (59.38 %) was observed in control treatment whereas minimum damage (0.56 %) was observed in grain treated with 10 % oil (Table 6.).

### 3.6 Germination percentage

Maximum germination was observed in grain treated with 10 % concentration (95 %) followed by 5 % (87.5 %) and 2.5 % (85 %) concentration, respectively (F_7, 32_ = 55.31, P < 0.001) (Table 6). The lowest germination (30 %) was observed in untreated maize (Table 6).

Similar alphabets within the column indicates no significant differences among the concentrations.

## 4 Discussion

Essential oil of plants can control pests, especially coleopteran [42]. These oils may be an effective alternative to chemical insecticides. *A. calamus* have the properties either to repel or can cause mortality to insect pests of storage and has extensively been used either in powder form or its essential oil against *Sitophilus oryzae* [18,19,43], *S. zeamais*, *Callasobruchus maculactus* [44], *Phthorimaea opercullela* [45], *Tribolium castaneum* and *Lasioderma serricorne* [21], *Spodoptera litura* [46]. Beta asarone [19,46,47], Shyobunone & isoshyobunone [22] and methyl eugenol & (E)-Methyl isoeugenol [48,49] are the major component present in *A. calamus* which showed toxic effect to insects.

In this study, sweet flag essential oil in scintillating vial bioassay was found to cause mortality in short period of time with low toxicity. High mortality with short survival was probably due to the combined effect of contact as well as fumigant effect inside closed scintillating vial. The fumigant and contact effect of the essential oil against *S. zeamais* adults was tested and described by various researchers [12,20,24], where small volume of concentration can cause higher mortality.

Our results showed that 2.92 % oil at 3 h and 8.75 % oil at 24 h were required to cause 50 % and 90 % mortality in scintillating vial bioassay test. Aryal et al. [50] reported the LC_50_ value of 36.8 and 36.4 ppm of sweet flag ethanol extract of Nepalese and Korean origin respectively to newly emerged potato tuber moth. Liu et al. [48] showed the stronger contact toxicity of *A. calamus* with LD_50_ value of 100.21 μg/cm^2^ to book lice *(Liposcelis bostrychophila)* than other essential oil. Hossain et al. [51], reported the isolated compounds, shyobunone and isoshyobunone also exhibited strong contact toxicity against *S. zeamais* adults with LD_50_ values of 20.24 and 24.19 μg/adult, respectively. The component such as shyobunone and isoshyobunone derived from *A. calamus* essential also exhibited strong contact toxicity with LD_50_ value of 61.90 μg/adult for *Tribolium castaneum* [21].

Median lethal time for the 10 % oil to cause 50 % mortality of weevil in scintillating vial bioassay was less than an hour while for the lowest concentration of 0.16 %, it was estimated about 28 h. Likewise, highest three concentration cause 100 % mortality of weevil within 12 h of time in our study. Kim et al. [12] elucidated that the 100 % mortality of weevil could be achieved within 3 days of contact with *A. calamus* oils to *S. oryzae,* when treated in an area 3.5 g/cm^2^ on filter paper. However, Park et al. [19] reported 100 % mortality occurred only after 7 days for *S. oryzae* but 100 % mortality for *Callasobruchus chinesis* was obtained at 3 days after treatment. Sukla et al. [52] found that 100 % mortality of *C. chinensis* could be achieved with 0.01 μl/ml of essential oil of sweet flag after 24 h of treatment as fumigant. Variable mortality may depend upon the different species of *Acorus*, active compound present in the essential oil and the species of test insect itself [53].

While evaluating the toxicity of oil on treated maize, we found that LC_50_ value at 6, 10 and 16 days were 2.89, 0.26 and 0.07 % respectively. We observed that, when exposure period is more, there was a progressive increase in the toxicity of the oils to the test insect showing appreciable mortality of *S. zeamais*. De Carvalho et al. [24] tested contact effect of essential oil against *A. calamus* where he found LC_50_ of 0.61 μl/20 g of beans. When maize grains were treated with 0.01 % oil and tested against *Acorus* sp., it significantly reduced the grain loss for 21 days of the treatment [21]. El-Naha et al. [54] reported that the period of exposure is crucial factor to cause mortality by *A. calamus*. They also found that oil caused mortality and also restricted feeding strongly up to 50 % within 48 h of treatment. Our results were in agreement with the findings of Sukla et al. [52], who concluded that the dose of 5 mg rhizome powder of *Acorus calamus* proved fatal, causing 100% mortality. Paneru et al. [18] reported that 2 % w/w *Acorus* rhizome powder admixed with maize grain kill all the test insects (*Sitophilus oryzae and Sitophilus granarium*) within 7 days of exposure. The 100 % mortality of *C. maculatus* at 0.09 and 0.1 % concentration of sweet flag extracted with hexane was obtained after 5th day of treatment [55] but 100 % *S. zeamais* mortality in our study was obtained with three highest concentrations within 24 days after treatment applied.

Repellent properties of plant essential oil to different insect pests has been studied by various researchers [20,22,37,52,56]. Our result indicated that sweet flag essential oil showed significant repellent effect to *Sitophilus Zeamais*. In our research, lower concentration of 1.25 % oil showed 77.5 % repellency and higher concentration of 10 % oil showed 98.75 % repellency after 24 h’ exposure. Similar results were observed in the research conducted by Chen et al. [22], where up to 98 % repellency was noticed when essential oil was tested against *Tribolium castaneum.* Repellent action was also highly dependent upon essential oil concentration, exposure time and host insects. Sukla et al. [52] conducted Y tube repellency test where they found that 150 μl of essential oil of sweet flag repel 97.3 % of *C. maculatus*. In another study by Yao et al. [20], repellency of 93.92 % was achieved for *S. zeamais* after 12 h when filter paper was treated at 629.08 μg/cm^2^. Similar results were presented by Kafle and Shih [38] where 97 % of *Solenopsis invicta* repellency could be achieved with *A. calamus* within 18 h of treatments. These differences in repellency potential may be due to extraction procedure employed, active compound present in essential oil or test insect species. High repellency even with lower concentration has significance value in reducing grain loss in storage condition. Currently used fumigants such as phospine could be highly toxic to humans as well as non-target organisms. As essential oil is of biological origin not only play a vital role replacing toxic chemicals to save the grain, and seed, but it also saves environment.

Essential oil of sweet flag helps to reduce the weight loss in maize by *S. granarius* [21]. Our study reveals that the weight loss in untreated control was nearly 4 times higher than lowest concentration tested (0.156 %). Likewise, concentration of 10 % oil reduce the damage by 4 times higher than the lowest concentration. The ability of the essential oil to prevent seed damage and weight loss could be due to early insect mortality. In our study, essential oil exhibited inhibitory potential for ovi-position of *S. zeamais* in all the concentrations as there was no progeny emerged. Similar result was obtained by Saranya et al. [44], where they found no progeny production at highest concentrations (0.05, 0.06, 0.07, 0.0.08 and 0.09) of sweet flag essential oil, which was tested against *C. maculatus*. Not only doses but also increase in exposure time also have effect upon progeny reduction which was shown by Schmidt et al. [57]. Increase in damage percentage for the grain treated with low concentration oil may have adverse effect on the embryo of the grain which ultimately hinder germination. This clearly showed that the essential oil of *A. calamus* either cause mortality or can repel maize weevil with low concentrations thus can be used as a component of integrated pest management.

Death of the insects may be due to the inability of insect to detoxify β-asarone as a result of P450 genes inhibition [40]. Similarly, genotoxicity have been reported by Kumar et al. [58], where damage of gene is the cause of mortality in *Dorsophila melanogaster*. Essential oils of plant have effects on plasmocytes and granular hemocytes. Thus, destruction of these organelles, insect lose their defense mechanism to counter act the toxic effect of essential oil [59]. Ovi-position inhibition may be due to the early death of the female or some behavioral and physiological changes in adults due to essential oil. Essential oil of *A. calamus* supposed to cause male sterility due to the sperm ailment like agglutination and immobility of sperm in male as well as the sperm in female tracts are also changed greatly in morphology [60].

Some isomers of the asarone are said to be carcinogenic and possess cardiotoxicity, hepatotoxicity, reproductive toxicity, mutagenicity at varying levels [61,62]. This toxicity depends upon the amount of daily exposure. Therefore, there are restrictions and rules enforced by various authorities on the use of extracts of *A. calamus*. LD_50_ of essential oil of sweet flag has set to 350 mg/kg body weight [63]. *Acorus calamus* oil and its extracts are prohibited from use in food in US. However, the European Council adopted the limit of β asarone with the amount of 0.1 mg/kg and 1 mg/kg in food stuff and alcoholic beverages, respectively as flavoring and seasoning ingredients [64]. Since availability of the tetraploid *A. calamus* in the Asian region is prevailing which contain β asarone in highest amount, use of essential oil from *A. calamus* of this region must be regulated and its decomposition rate and residue should be investigated after certain period of application.

## CRediT authorship contribution statement

**Sunil Aryal:** Conceptualization, Data curation, Formal analysis, Methodology, Supervision, Writing – original draft, Writing – review & editing, Project administration. **Ashmita Poudel:** Data curation, Investigation, Writing – original draft, Project administration, Writing – review & editing. **Kapil Kafle:** Methodology, Supervision, Visualization, Conceptualization, Writing – review & editing. **Lok Nath Aryal:** Formal analysis, Validation, Writing – review & editing.

## Declaration of competing interest

The authors declare that they have no known competing financial interests or personal relationships that could have appeared to influence the work reported in this paper.
